# Ultrasound and sonochemistry enhance education outcomes: From fundamentals and applied research to entrepreneurial potential

**DOI:** 10.1016/j.ultsonch.2024.106795

**Published:** 2024-02-06

**Authors:** David Fernandez Rivas, Pedro Cintas, Jarka Glassey, Daria C. Boffito

**Affiliations:** a Mesoscale Chemical Systems Group, MESA+ Institute and Faculty of Science and Technology, University of Twente, P.O. Box 217, 7500 AE Enschede, the Netherlands; b Departamento de Química Orgánica e Inorgánica, and IACYS-Green Chemistry & Sustainable Development Unit, Facultad de Ciencias-UEx, 06006 Badajoz, Spain; c School of Engineering, Merz Court, Newcastle University, Newcastle upon Tyne NE1 7RU, UK; d Department of Chemical Engineering, Engineering Process Intensification and Catalysis (EPIC), Polytechnique Montréal, C.P. 6079, Succ. “CV”, Montréal H3C 3A7, Québec, Canada; e Canada Research Chair in Engineering Process Intensification and Catalysis (EPIC), Polytechnique Montréal, C.P. 6079, Succ. “CV”, Montréal H3C 3A7, Québec, Canada

**Keywords:** Cognitive models, Chemical engineering, Educational tools, Sonochemistry, Process intensification, Entrepreneurial education, Scale-up

## Abstract

With this manuscript we aim to initiate a discussion specific to educational actions around ultrasonics sonochemistry. The importance of these actions does not just derive from a mere pedagogical significance, but they can be an exceptional tool for illustrating various concepts in other disciplines, such as process intensification and microfluidics. Sonochemistry is currently a far-reaching discipline extending across different scales of applicability, from the fundamental physics of tiny bubbles and molecules, up to process plants. This review is part of a special issue in *Ultrasonics Sonochemistry*, where several scholars have shared their experiences and highlighted opportunities regarding ultrasound as an education tool. The main outcome of our work is that teaching and mentorship in sonochemistry are highly needed, with a balanced technical and scientific knowledge to foster skills and implement safe protocols. Applied research typically features the use of ultrasound as ancillary, to merely enhance a given process and often leading to poorly conceived experiments and misunderstanding of the actual effects. Thus, our scientific community must build a consistent culture and monitor reproducible practices to rigorously generate new knowledge on sonochemistry. These practices can be implemented in teaching sonochemistry in classrooms and research laboratories. We highlight ways to collectively provide a potentially better training for scientists, invigorating academic and industry-oriented careers. A salient benefit for education efforts is that sonochemistry-based projects can serve multidisciplinary training, potentially gathering students from different disciplines, such as physics, chemistry and bioengineering. Herein, we discuss challenges, opportunities, and future avenues to assist in designing courses and research programs based on sonochemistry. Additionally, we suggest simple experiments suitable for teaching basic physicochemical principles at the undergraduatelevel. We also provide arguments and recommendations oriented towards graduate and postdoctoral students, in academia or industry to be more entrepreneurial. We have identified that sonochemistry is consistently seen as a ‘green’ or sustainable tool, which particular appeal to process intensification approaches, including microfluidics and materials science. We conclude that a globally aligned pedagogical initiative and constantly updated educational tools will help to sustain a virtuous cycle in STEM and industrial applications of sonochemistry.

## Highlights

• Sonochemistry in education builds valuable physico-chemical fundamentals.
• Sonochemistry case studies reinforce process intensification knowledge.
• Sonochemical case studies aid in building academic and industry-oriented careers.
• A global initiative can sustain a virtuous cycle in STEM and entrepreneurship.
• Sonochemistry supports STEM outcomes with added entrepreneurial skills.

## 1 Introduction and Motivation

**Fig. 1:**
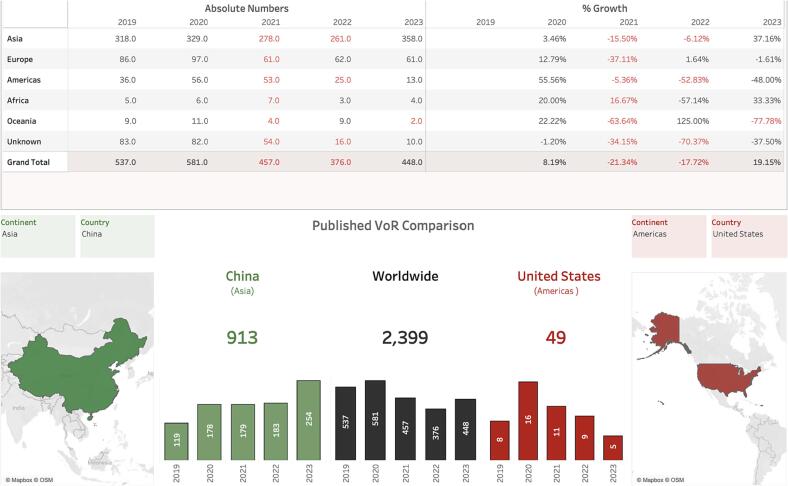
Total number of articles published in Ultrasonics Sonochemistry over the last five years, organized by continental areas. The two main contributing countries are China (9 1 3) and United States (49), representing Asia and the Americas respectively.

Sonochemistry involves multiple applications of ultrasound waves in chemical processes and engineering, which has gained increasing and impactful popularity in recent decades. Dating back to the late 1920 s when tycoon Alfred Loomis and associates performed the first ultrasonic experiments, without realizing the actual mechanisms involved [1], [2], modern sonochemistry with systematic exploration in organic and organometallic chemistry, can be traced to research conducted from the early 1980 s onwards. Since then, the interest and range of interdisciplinary applications have grown impressively. One plausible reason of this popularity is the accessibility, relative affordability, and low volume footprint of ultrasonic equipment. Different types of ultrasonic equipment are now in use in laboratories worldwide, arguably making it one of the most popular tools available, see Fig. 1. Ultrasonic processes tend to be safe or involve only minimal operational risks, which has lowered the entry-barriers to users outside the traditional academia, also facilitating their geographical spread. For example, most sonochemical processes are overall safe to be manipulated even by not-so-experienced operators, without chemical fume hoods.

**Fig. 2:**
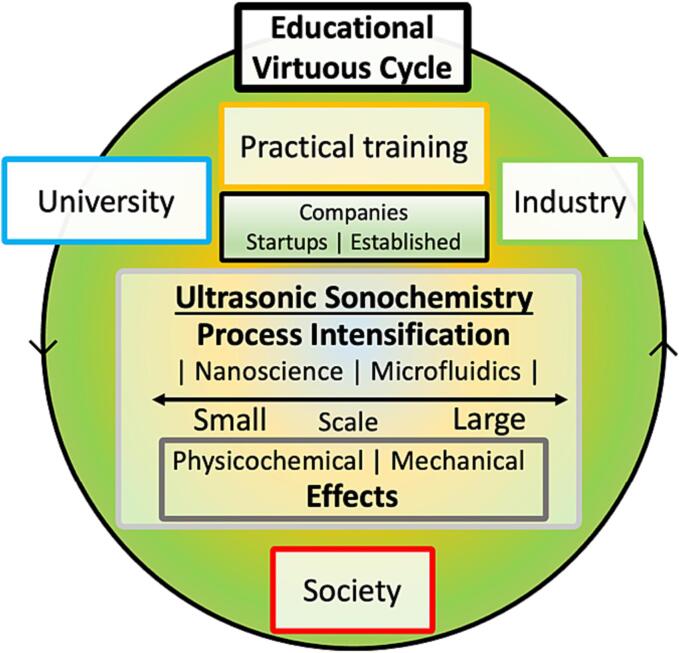
From physical fundamentals to applied chemistry, ultrasound pervades the intertwined domains of education, basic research, industrial pursuits, and everyday science. Ideally, this indicates a virtuous cycle to inspire all stakeholders, which spins around innovation at different spatiotemporal dynamics, including process intensification, microfluidics, and nanoscience.

From an educational viewpoint, ultrasound-based applications are unique in combining the concepts of *ultrasonics*, a subdiscipline within the physical field of acoustics, with *sonochemistry*, addressing chemical-oriented issues in broad sense. This uniqueness enables both feedback and feedforward along different personal training and working scales and the interest of different stakeholders, from academia to the industry (Fig. 2). Not by chance, this underlying combination matches appropriately the name of this journal.

The broad use of ultrasonics sonochemistry can be linked to its multidisciplinary adaptability, including very diverse domains. The list is long, and includes *bubble formation and cavitation mechanisms, chemical synthesis and processing, fabrication of materials and nanomaterials, biomass valorization, waste degradation and recycling, food processing, sonocatalysis and piezocatalysis, reactor design, microfluidics, ultrasonic sensors, crystallization, extraction, mechanochemistry, polymer formation/degradation, drug delivery, sonodynamic therapy and therapeutic focused ultrasound,* etc. The number of articles and reviews documenting a range of applications is countless. Newcomers and interested readers in general are, however, referred to early monographs, where some pioneers delineated the background and newer applicability of ultrasonic activation, including experimental guidelines [3], [4], [5], [6], [7]. More recent titles and comprehensive treatments pay attention to further developments and emerging applications [8], [9], [10], [11], [12], [13].

**Fig. 3:**
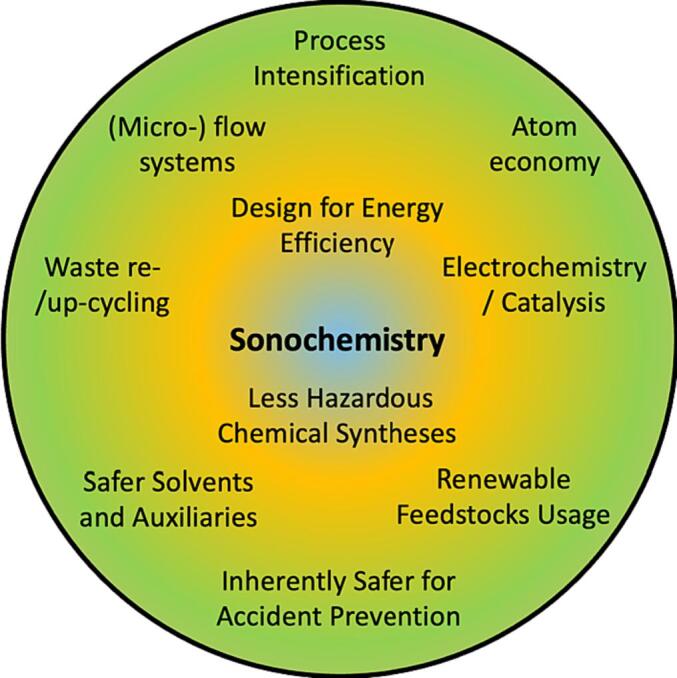
Examples of research domains where sonochemistry can synergize with a selection from the 12 design principles of Green Chemistry principles, electrochemistry and photocatalysis.[19], [20], [21]. (For interpretation of the references to colour in this figure legend, the reader is referred to the web version of this article.)

A clear trending shift is the merging of sonochemistry with green chemistry and green engineering, thus joining the suite of enabling technologies. These enabling technologies comprise electrochemistry, photochemistry, mechanochemistry, microwaves, plasma, high pressure, and others aimed at creating a more sustainable world. Education in sustainability is now almost universally compulsory [14], [15], not only to preserve our fragile planet’s finite resources, but also to meet the sustainable development goals of the United Nations (UN-SDG) [16] to eradicate poverty, protect the environment and improve the quality of life [17], [18]. Certainly, ultrasound can be applied efficiently (if well designed) and safely to current challenges involving our limited and renewable resources and using less-hazardous protocols (Fig. 3). Traditionally, ultrasound has been broadly used as advanced oxidation processes (AOP), where the cavitation effects add radicals, e.g., OH^.^, in a synergistic way to another AOP like photocatalysis or Fenton. Additionally, an ultrasonic step can be added to a unit operation (reaction, extraction, crystallization, distillation) to improve overall performance, such as clogging reduction and other mass transfer phenomena.

Sonochemistry can contribute to the “Atom economy” by promoting contactless mass transfer during a process to facilitate the incorporation of all chemical entities into the final product. As ultrasound-assisted syntheses often require a lower solvent inventory, catalysts, and initiators, sonochemical syntheses are often “Less hazardous chemical syntheses” and conventional reagents can be replaced by “Safer solvents and auxiliaries”. When well designed, sonochemistry can be integrated in selected commercial processes to promote “Design for energy efficiency” as the energy of the mechanical waves delivered to processes with macro-shear mixing and/or acoustic cavitation can decrease the overall energy requirements [21], [22]. “Use Renewable Feedstock” presents several challenges as renewable feedstock is often either physico-chemically heterogeneous or target molecules are found diluted in a matrix. Once again sonochemistry can help disintegrate the outer matrix to release these interesting molecules and accelerate the processes to transform renewable feedstock into valued chemicals [23], [24], [25], [26]. Finally, as ultrasound-assisted processes use less solvents and auxiliaries or allows these later to be replaced with safer ones, and energy requirements are often minimized, “Inherently safer chemistry for accident prevention” is another green chemistry trait that can characterize sonochemistry. For example, during the collapse of bubbles, very high temperatures and pressures are reached inside the bubble without changing the liquid macroscopic parameters.

**Fig. 4:**
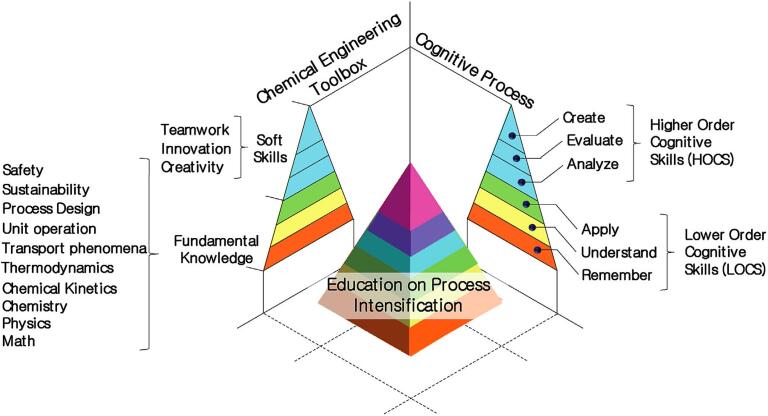
Visual integration of basic and applied concepts in chemistry and engineering with cognitive elements towards education on process intensification. Reproduced from Reference [28], OA article under the CC BY license. Copyright 2020 by the Authors and published by Elsevier B.V.

In the last decades, we have witnessed an innovation trend, particularly around the term ‘intensification’. In terms of technological solutions and large-scale production, the engineering approach of process intensification is aimed at boosting efficiency and safety while reducing costs [27], [28]. Process intensification has matured as the integration of fundamental research, and as a result, chemical engineering is moving from a traditional focus on unit operations, process chemistry towards the inclusion of concepts such as chemical synthesis and materials design, catalysis, and alternative energy input as exemplified by ultrasound and electrochemistry [20], [29]. In a pedagogical context, we recently proposed a framework of process intensification and cognitive skills to help educating the future generations of chemists (Fig. 4) [28].

**Fig. 5:**
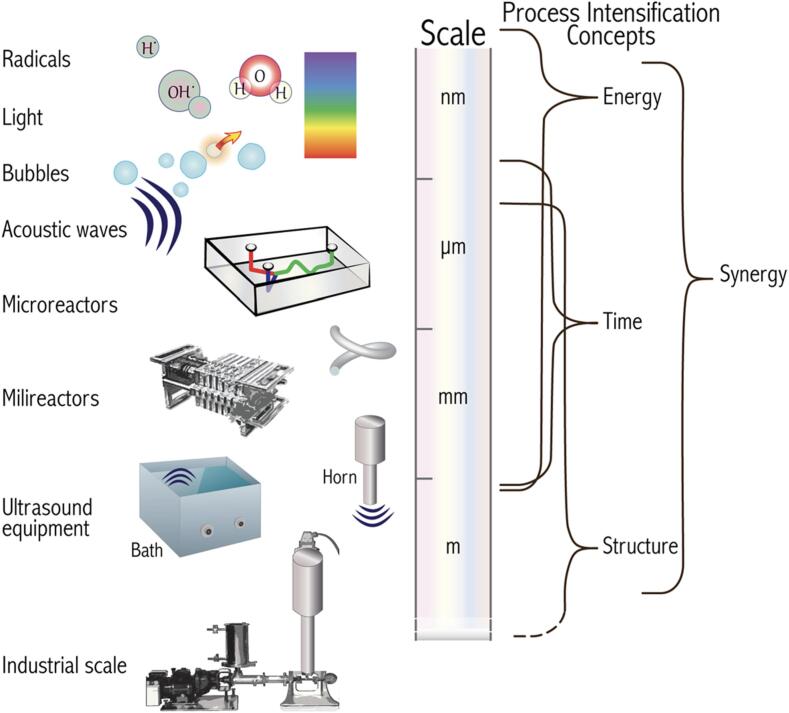
The relative sizes of the items described on the left increase from top to bottom. On the right we connect the different sizes where the process intensification concepts have the greatest influence. For educational activities, it is common to find equipment such as ultrasonic baths and horns that fit on a tabletop. Diagram with terms and concepts described in Ref.[34]. Reproduced with permission. Copyright 2016 by Springer.

It is now widely accepted that the term *greenness* should be used with caution unless one considers all variables involved in a synthetic design (solvents, separation, purification) [30], [31], along with life cycle assessments in process intensification from raw precursors to end products, including costs and risks [32], [33]. Nevertheless, compared with other enabling technologies aimed at UN-SDGs, ultrasonic devices, usually portrayed by the archetypal cleaning bath or horn, can be regarded as low-cost and easy-to-use equipment, see Fig. 5. This last figure highlights the difference in size scales, from miniaturization, exemplified by microchips, sensors or microfluidic devices to lab and pilot units, all embracing identical physical background, albeit facing different technical parameters [34].

Paradoxically, in many instances the exact role of sonication is poorly understood, or not understood at all. One reason is invariably linked to the complex nature of cavitation, a nonlinear phenomenon that depends on experimental variables with great variability, e.g., liquid temperature, gas content, glassware used. Next, the lack of appropriate knowledge and training in the technique and its fundamentals have led to a large number of publications that not always provide accurate or reproducible information [35]. Moreover, the improper use of statistical analyses and operation protocols of ultrasonic equipment complicates the identification and characterization of (ideally quantitative) activation-effect relationships. To a significant extent, numerous scientists who face the use and applications of an ultrasonic apparatus receive a crude message that can be summarized in no more than *“just switch on, collect data, and insert a few sentences/citations on cavitation when writing the introductory paragraphs”*.

On the other hand, sonochemical or ultrasound-guided experiments expose the modern aspects of this field to both undergraduate and postgraduate students as optimal solution for solving technical problems. Ultrasonic lab practices may also further deepen the understanding and applicability of such pressure waves. Thus, teaching and learning goals can be summarized in terms of (1) knowledge and subject cognition, and (2) experimental methods and skills. The purpose of this article (and the whole special issue) is both to raise awareness and to promote education in sonochemistry as an effective tool for (chemical) engineers and scientists in their efforts to develop sustainable solutions to grand societal challenges. The fact that ultrasonic equipment uses electricity can help in convincing of its relevance for ongoing electrification of the chemical industry, particularly when renewable sources can be utilized. Thus, this article was aimed at addressing some of the misconceptions around the sonochemistry concepts and highlight the means of incorporating sonochemistry concepts into the education of future generations of (chemical) engineers and scientists.

## 2 Illuminating the black box

### 2.1 On concepts and misconceptions

At the dawn of sonochemistry as an independent discipline, some experts warned about the pitfalls of reporting data without quantitatively assessing the acoustic scenario [36], a point of caution raised by acousticians too [37]. More than three decades later, the notion of sonochemistry as “black art” [36] still persists as reproducible results cannot be envisaged in the absence of precise data on the type of sonoreactor, flask geometry, or energy aspects. Optimization of such variables influences the outcome of ultrasound-based transformations as well as analytical protocols when sonication is applied to sample preparation, among others [38]. A random selection of a vast number of papers from, for example, the area of material/surface modification and nanoparticles, shows that experimental set-ups indicate no more than an unmodified commercial apparatus (not always identified as bath or probe, or reasons accounting for their usage), together with the nominal frequency and output power indicated by the supplier, which are largely meaningless. Even worse, since reviewers may not be familiar with sonochemistry, the importance of determining a few working parameters goes virtually unnoticed.

A tutorial on sonochemistry invariably will benefit from some basics of acoustics. Sound is essentially a *mechanical force* whose propagation through an elastic medium can be described by *pressure waves* involving compression and rarefaction cycles. Wave motion at all frequencies (including ultrasound > 20 kHz, and those for clinical diagnosis between 1 MHz and 10 MHz) can be interpreted by well-known wave equations (complex equations are required for nonlinear phenomena), and typical behaviors of reflection, refraction, absorption, and scattering are observed. Unlike electromagnetic radiation, however, sound has no quantum character (with the exception of *phonons*, a concept beyond the scope of this article). Accordingly, frequency and energy do not relate to each other in a linear relationship. This likely represents a major source of misconception and biases, by extrapolating the characteristics of light to sound.

As ultrasound passes through a medium, it transports energy. Unfortunately, the definitions of energy (in Joule), power (in Watt) and intensity (Watt/cm^2^) in the context of acoustics are not immediately obvious. Unlike the energy associated with a photon, one can only estimate the (average) energy of the acoustic field, which is informative at a given frequency. The rate of energy transport can be denoted as “power”. Since ultrasound is produced in beams that are focused on small areas, intensity can be described as power per unit area (and hence in W/cm^2^. Power and intensity are often misused as equivalents, and in the audible range, the terms sound power and intensity are usually interchanged and referred to as *loudness*. Lay people are likely familiar with the decibel scale, a logarithmic scale expressed as:

(1) $$\mathrm{dB}=10\log\left(I/I_{o}\right)$$

where I_o_ is the reference intensity. For audible sound this makes sense, because the accepted reference is I_o_ = 10^−16^ W/cm^2^, i.e. the lowest intensity the human ear is able to perceive. At this intensity, a 1 kHz tone (audible, musical note C) will hardly be distinguished. When intensity of such a note increases to 120 dB (=10^−4^ W/cm^2^), it will be extremely painful (an acceptable street-level noise in an urban environment should be below 60–70 dB). The problem with intensity is that it needs to be relative to some reference intensity, and no universal standard reference intensity exists for ultrasound and megasonic echography [39].

**Fig. 6:**
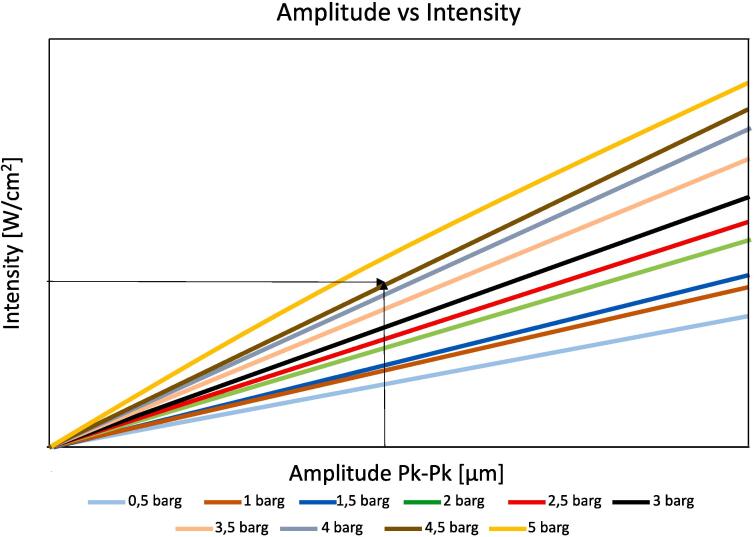
Example of calibration curves for amplitude (µm) vs intensity (W/cm^2^) of a commercial ultrasound emitter. Intensities and amplitudes vary with the type of ultrasound horn. Intensities can go up to 1000 W/cm^2^ and amplitudes may reach 200 μm in commercial ultrasound systems.

Ultrasound wave intensity could likewise be expressed in dB, albeit it is usually related to the maximum pressure of the wave (P_m_ or P_A_, i.e. acoustic pressure amplitude, denoted in Pascal) in the medium by:

(2) $$I=P_{m}^{2}/2rc$$

where ρ is the density of the medium (kg/m^3^) and *c* is the speed of sound in the medium (m/s). This simple expression is valid only for planar or spherical waves obtained within low-pressure changes. Cavitation at low-frequency leads to significant changes in acoustic pressure, which translates to a nonlinear behavior. In practice, the intensity ratio of two waves can be related to their amplitude ratios. In modern equipment, the output voltage of the transducer will determine the vibration amplitude. Therefore, the efficiency of a sonochemical process can be compared at different vibrational amplitudes. That said, things are not quite so simple, because another common misconception is to equal power and amplitude although they are linearly dependent. The former, typically referred to as “nominal power” is the maximum power at which a transducer power output (TPO) can drive a piezoelectric transducer (which should also be designed to withstand such power). The power delivered to a process can usually be fine-tuned by choosing % of the maximum nominal power on the instrument controller. Amplitude is the maximum extent of the sound wave, typically measured from the baseline to the highest point. For over-the counter sonochemical devices, amplitude can go up to several hundred of micrometers (µm) and it typically means that higher powers correspond to higher amplitudes. Manufacturers should provide a calibration graph of amplitude vs. power for a given device (an example is given in Fig. 6). Indeed, two different ultrasound devices working at the same power may have been designed to emit different sound amplitudes. Regardless, in a liquid medium, the power and the amplitude of an ultrasound wave can undergo changes based on several factors, including attenuation, absorption, scattering, etc., mainly dependent on the properties of the liquid and the characteristics of the ultrasound wave.

The operation frequency of a sonochemical experiment matters very much. Similar to the considerations of the acoustic field above, comparable effects can be obtained within a range of ultrasonic frequencies. For educational purposes, students and newcomers are first instructed in the use of single-frequency devices and the shape and dimensions of the emitter (probes in particular) should be specified. There are frequency effects because cavitation hinges not only on the reactive species produced after bubble collapse, but also on gas content, and on the bubble lifetime and radius, which are modulated by the acoustic frequency [40], [41]. In the usual range of sonochemistry, low frequency (20–100 kHz) and high power are applied, and mechanical effects will be prevalent. Contrarily, above 100–200 kHz and up to MHz frequencies, chemical effects associated with enhanced radical production represent the dominant mechanism [42]. Irrespective of a clear-cut knowledge of cavitational phenomena, students grasp well that as the compression-rarefaction cycles shorten, bubble implosion becomes less violent and hence, a greater intensity will be required to make the liquid cavitate at high frequencies.

We strongly encourage the Ultrasonics Sonochemistry community to work towards a General Introduction to the use of basic equipment. This common starting point will surely contribute to reaching a ‘common language’ or understanding across nations, sub-disciplines and a faster adoption by industry and startup companies.

### 2.2 Measure for measure

At this stage, it could be intimidating to ascertain how to control a sonochemical experiment. For numerous applications, chemical synthesis in particular, for which sonication could alter the reactivity and product distribution relative to a silent protocol [43], it suffices to establish a few working conditions [44]. It makes no sense to worry about a specific frequency for instance, because the resonance frequency of an ultrasonic apparatus may be modified by other effects, such as temperature or the total liquid volume inside the reactor bath. In the early 1980 s, the late acoustician Robert Apfel pointed out that all significant problems in acoustic cavitation (the term *sonochemical* was introduced in 1980 [45]) could be related to the ignorance of the acoustic field [46].

**Fig. 7:**
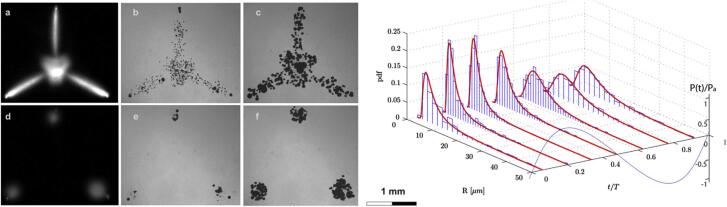
(Left pane) Images of a substrate with three artificial bubble nucleation points at different powers and at two selected points in the acoustic cycle. The upper row corresponds to high power (0.981 W) and the lower row to low power (0.194 W) with normal illumination conditions (a and d) and corresponding short exposure images: b and e in the compression; c and f in the expansion phase of the acoustic cycle. (right pane) Corresponding bubble size distribution histograms; the axis to the extreme right represents the normalized pressure for the acoustic cycle. Adapted with permission from [47].

Apfel formulated three key tips in a Socratic style, now known as *Apfel’s golden rules*, which can be summarized as: (1) “know thy liquid”, (2) “know thy sound field”, and (3) “know when something happens”. The notion of cavitation in liquids indicates that the solvent is much more than just a medium to dissolve reactants and reagents and that it affects both sound transmission and bubble implosion. Thus, the first rule bears in mind the importance of the liquid properties (vapor pressure, viscosity, surface tension, or bond strength) to determine the cavitational threshold. The second rule focuses on the accuracy of working parameters, while the last precept suggests that observable effects could indirectly inform about the extent of cavitation in the reaction system. Collectively, such tips highlight the significance of acoustic parameters and the proper use of ultrasonic devices. The third rule was difficult to follow in detail until the advent of faster cameras coupled to microscopy, and more precise equipment, such as optical fiber hydrophones. These new technologies, enhanced by faster and powerful computer technology, enabled a better understanding of what happens at the smallest scales and high temporal frequencies driven by ultrasound. Imaging shockwaves and swarms of bubbles jetting, emitting light or interacting with objects infused the ultrasonics sonochemistry field with new ideas and opportunities, see Fig. 7.

The usual way to characterize the energy delivered into a reactor, generally using either a cleaning bath or an ultrasonic horn, involves the estimation of the transmitted power. This value can be obtained from a combination of physical and chemical measurements, which represent the core of dosimetry methods. Moreover, students’ learning is greatly facilitated through dosimetry by integrating the notion of cavitation with methods in chemical analysis, thermodynamics, and reaction monitoring. The easiest physical dosimetry implies the assimilation of the reaction vessel to a calorimeter (including the ultrasonic tank) and plotting the changes in temperature versus time. Beyond the cavitation threshold, the acoustic energy is dissipated partially into heat. The calorimetric power (in Watt) can be roughly estimated from the equation [3], with slope ΔT/Δt:

(3) $$P\left(w\right)=m\cdot Cp\cdot \Delta T/\Delta t$$

where *m* is the mass of liquid (expressed in g) irradiated with ultrasound and *Cp* the isobaric thermal capacity (in J/g·K, if slope is expressed in K/s). It is obvious that the thermal variation across the liquid could not be spatially homogeneous and therefore, sonication of small volumes is desirable to achieve reproducibility.

Chemical dosimetry is based on the quantitation of radical intermediates generated by sonolysis, or end products, by means of UV–Vis or fluorescence spectroscopies, among others. In aqueous solutions, the formation of OH radicals that trigger subsequent oxidation of analytes constitute expeditious and visual illustrations accounting for the extent of cavitation. Reinvestigation of some popular dosimetries unveils disparate results and hence, comparative assessment is required for accuracy [48], [49]. More recently, a temperature independent HO· dosimetry using Res-DHB, as a highly selective fluorescent probe was proposed [50]. Differences can be ascribed to the fate of radical species, as the major amount likely recombines within the bubbles prior to collapse and only a small fraction could initiate further radical or redox reactions in solution. In principle, any method capable of quantifying a given species of interest (e.g. for environmental or atmospheric monitoring) could be employed as sonochemical dosimetry [51].

However, when radicals other than HO· are involved, electron paramagnetic resonance (EPR) seems to be the only tool that can be used to both qualify and quantify these ultrasonically generated radicals with the aid of spin-traps. Further, this method can be used also in the presence of solid media present in different granulometry in the sonicated system [52]. The main limitations of EPR is the high cost of the spin trap chemicals (e.g. stable aminoxyl radicals like TEMPO and DMPO) as well the difficult interpretation of the spectra involving overlapping signals, deconvolution, etc. [52]. Except EPR, all these dosimetry methods are relatively easy to implement, and thus inexperienced students and researchers may relatively effortlessly put them into practice and calculate the sonochemical yield in different systems and compare it for either different ultrasound devices (in the same vessel) or different unit geometries (with the same ultrasound emitting unit). Accordingly, chemical dosimeters are highly formative and instructive in sonochemistry courses, and numerous physicochemical concepts can be reinforced as well. A detailed discussion, in the context of pedagogical tools, is provided later (see Sect. 3).

What matters, in line with the third Apfel’s rule, is to capture a distinctive effect that can be measured when the system is activated by sonication. Multiple cavitation effects, both physical and chemical in nature, may occur when pure liquids or heterogeneous systems are irradiated, even though such intertwined phenomena will hardly be disentangled. We contend that the more details we know about the acoustic field, the more we can define it and make the experiment more reliable and reproducible.

However, one should recognize that all dosimetry methods offer a relative measure of the acoustic activity in a system. Although they are quite easy to perform, when the aim is to determine the physicochemical impacts of acoustic cavitation in a complex system, the system may contain different phases and species. A combination of different dosimetry methods and characterizations is key to have a deeper understanding of the outcomes induced by ultrasound.

**Fig. 8:**
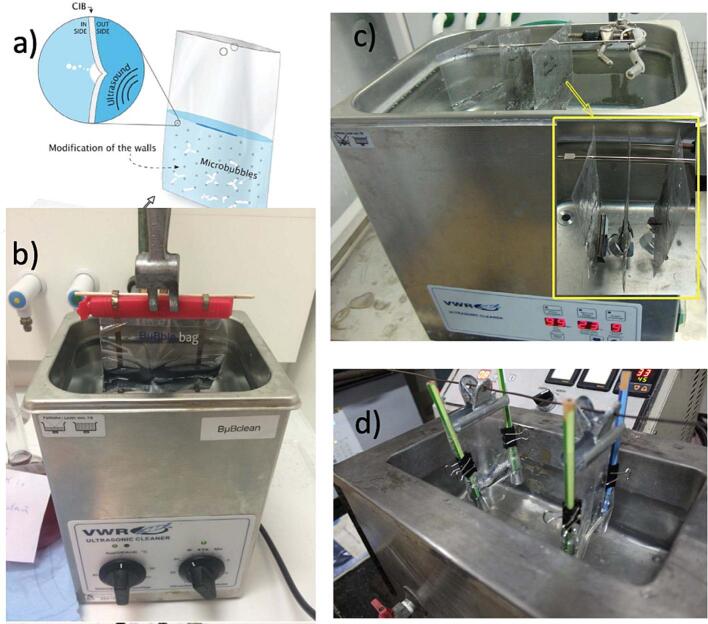
a) Schematics of a Cavitation Intensification Bag (CIB) inside an ultrasonic bath (adapted with permission from [54]). b) Experimental setup used for the samples prepared in Enschede, The Netherlands; b) two bags are fixed inside the ultrasonic bath in Mumbai, India and c) Kuopio, Finland with inset showing the position of the CIB.

Adapted from [35]

We present an example of how the widespread usage of ultrasonic equipment can contribute to resolving one of the biggest challenges of scientific research in sonochemistry in particular. Reproducibility of results across laboratories has always been difficult, and if we want to use sonochemistry in education of younger generations effectively, clear examples of reproducibility are required. The disparate types of equipment used, from manufacturers of the electronic parts to glassware, and different lab protocols all led to the notion that it is impossible to replicate results. To address this challenge, we present the case where experiments were independently carried at three locations, India, Finland and The Netherlands. The three groups used a novel type of reactor, the Cavitation Intensifying Bags [53], which is an example of a scaled–up sonochemical microreactor with increased efficiency and reproducibility [35], see Fig. 8.

The study had illustrative applications: potassium iodine (KI) liberation, methyl-blue (MB) degradation; and exfoliation of two nanomaterials, graphene and molybdenum disulfide [35]. The reproducibility of these experiments was compared to previous experimental results under similar conditions. The study concluded that the complexity of cavitation as a nonlinear phenomenon whose quantitative estimation represents a challenging aspect can be tackled with a list of procedural steps that can help improving reproducibility and scale-up efforts:

1. *“Scale-down” first to understand the phenomena at stake. Particularly from the fundamental physicochemical causes and effects of cavitation.*
2. *Try to control and number up sources of cavitation (passive or without more energy input).*
3. *Keep in mind that more “power” or “pits” does not necessarily yields the best results.*
4. *If possible, try to optimise the geometry, and other parameters that influence most physicochemical properties.*
5. *When needing larger production volumes, try and scale-up by using materials that are relevant to industry, easy to manufacture, and adapt to specific settings (*e.g. *cleaning of filtration membranes, food processing industry, medical cleaning, etc.).*
6. *If trying to translate or valorise scientific results and deploy into society, look for good industrial and scientific partners that can help minimizing resources and time consumption*.

The study proposed a final call to the multidisciplinary community of sonochemists: “we are in favor of a joint initiative in which an electronic handbook of sonochemistry experimental analysis can be assembled, edited, and regularly updated by researchers active in this field. Such handbook will serve not only newcomers to the field but improve the toolbox of more experienced researchers. It could be seen as a sonochemical database, describing experimental procedures after independent verification, as well as tips, safety recommendations, etc”. Such community-maintained initiative, akin to Wikipedia pages, could comprise information as shown on Table 1.

**Table 1:** Comparison of different experimental cases studied with Cavitation Intensifying Bags (CIB) and Normal Bag (NB), taken from [35].

Experiment	Dosimetry: Terephthalic Acid	Dosimetry: Potassium Iodine and Methylene Blue (MB)	Exfoliation: graphene	Exfoliation: Molybdenum Disulfide	Emulsions: C_16_H_34_ & Sodium dodecyl sulfate**
Location	Enschede, NL	Mumbai, India	Kuopio, Finland	Enschede, NL	Wageningen, NL
Temperature (K)	294	305	293	294	293 & 354
Frequency (kHz)	35 & 45	40	45	45 & 50–60 (horn)	37 & 80
Main result (CIB vs NB)	33 % smaller standard deviation + 45.1 % more efficiency	Higher initial rate of iodine liberation and MB degradation in CIB	Improved dispersion & open layer structure	High polydisperse layer sizes	60x more effective breaking up droplets
Reproducibility	+	∼	+	∼	+

+ or ∼ stands for increased reproducibility (+), or similar (∼) comparing the use of CIB vs NB. * Taken from [53], ** Taken from [54].

### 2.3 Ultrasonic equipment: tips from a hitchhiker’s guide

Training in sonochemistry does necessarily involve a primer on lab equipment. For nearly six decades, sonochemistry as discipline was virtually non-existent, but some scholars encouraged the use of sound and ultrasound waves in higher undergraduate education [55]. The authors highlighted analytical applications to measure the velocity and attenuation of sound in gases and liquids, or the design of ultrasonic nebulizers. Interestingly, gas chromatography with sonic detectors was also reported. Chemical effects were however discussed only briefly in terms of liberation of free chlorine from CCl_4_ upon irradiation in the presence of gases and stating that “*perhaps the true explanation of the phenomena described involves the influence of the gas on the violence of collapse of cavitation bubbles*”.

Despite theoretical considerations about acoustics and cavitation, ultrasonic apparatuses, exemplified by the common and ubiquitous cleaning bath and the most sophisticated, yet commonly available, ultrasonic horn, are easy to use and can be implemented in numerous practical activities at different teaching levels. Experiments can be rationally designed, and various adjustments may be required, such as the use of external bubbling gas, pressurized vessel, or external cooling. Some devices enable pulsed irradiation in order to provide accurate thermal control. Consistent with the aforementioned premises of “characterizing the acoustic field”, other considerations can be found in the literature [3], [6], [43].

Although the typical horn-based and ultrasound bath emitters in cylindrical-type of reactors are the most ubiquitous, there are other emitters and reactors of possible geometries [29]. For every geometry the link between power, power density and intensity, as well as other process variables should be characterized, ideally by computational fluid dynamics (CFD) first, to identify which reactor is most suited for a certain application. These different parameters make the ultrasound fall in different category, such as unfocused transducers, focused transducers; each suitable for different applications, from ablation (HIFU) to cleaning.

In general, sonochemistry is less hazardous and safer than other non-conventional energy sources such as microwave irradiation or UV photochemistry, making it more readily implementable in various teaching environments. The safety precaution measures, such as ear protectors and silent boxes, eyeglasses, and gloves, provide a relatively low cost means in comparison with the potential harm to humans. Adding to the reproducibility concerns discussed in the previous section, it would be helpful to create standard or universal protocols and make them openly available and ensure safe operations worldwide.

### 2.4 Sonoprocesses, scale-up and selected commercial applications

There are a few over the counter ultrasound reactors, ranging from laboratory to industrial scale, in batch and flow-through, as illustrated in Fig. 5. Shortcomings on scaling up sonoprocesses have been recently highlighted [29]. In a nutshell, different applications benefit from different frequencies, and these are in most cases well above the typical over the counter 20–40 kHz range. In addition, every system should be thoroughly characterized in terms of resonance frequency, acoustic pressure profiles, heat dissipation, radical concentration, fluid dynamics and fluid velocity profiles to maximize the beneficial effects of sonochemistry and scale-up ultrasound-assisted reactors with similar acoustic pressure profiles and acoustic activity as at the laboratory-scale. These aspects are highly dependent on reactor geometry, temperature, pressure, physicochemical properties of the medium, etc., which should be established at the laboratory scale and again at in the scaled-up reactor. Modeling techniques that describe different phenomena enacted by ultrasound are most useful to save time and resources in laboratory experiments and above all when scaling up a process (more tips on modeling are given in the next section).

However, a comprehensive modeling can be lengthy and burdensome. For a rough scale-up estimate of the size of an ultrasound reactor, two quick approaches can be adopted:

*Approach 1* – Linear scale-up: the first step in this approach consists of collecting data at the laboratory scale (usually with ultrasound processors operating at maximum nominal power ranging from 100 to 500 W) with volumes ranging from a few mL to 2 L maximum, in batch vessels. A design of experiments may be key to identify optimal operating variables, in particular power per unit of volume that maximize the output (usually conversion or selectivity) [56]. The second step in this approach consists in merely translating the same power required at the laboratory scale to achieve the desired output (on a W L^-1^ s^-1^ or W/g.s basis) to the bigger scale. This system does, however, lack finesse as it disregards the different acoustic pressure and cavitation distribution in the larger scale reactor, which is very likely of a different geometry compared to the the laboratory scale one.

*Approach 2* – Amplitude based: the same amplitude (in µm) should be transferred to the larger scale system to expect the same mass-transfer effects at the very vicinity of the tip, where the power and amplitude are the highest. The scale-up reactor should be designed as a flow system to maximize the power per unit of volume thus maximizing the exposure of the fluid being processed to this amplitude. Indeed, the ultrasonic power applied to the liquid load, and consequently the scale of the ultrasonic process, is determined by the surface area of the horn in contact with the liquid at a specific amplitude, pressure, and type of liquid [57]. The main limitation of this approach lies in the fact that the reaction mixture might need to be recirculated several times in the ultrasound chamber to achieve the same outputs as at the laboratory scale.

Several examples of scale-up of ultrasound reactors to commercial scale have already been highlighted [7], [29]. However, we are aware of a few other pathways which do not fit necessarily in the two approaches presented before. One is bringing ultrasound to the place where the effects are needed, such as ultrasonic reactors installed in a horizontal well for remediation of PFAS-contaminated groundwater [58] commercialized by RemWell [59]**.** Another approach has been controlling the nucleation process at the microscale, and a subsequent geometrical scale-up to provide a novel container or reactor vessel, see BuBble Bags in Fig. 8, commercialized by BuBclean [60], [61].

Other successful cases of large-scale applications, which otherwise highlight the opportunities provided by ultrasonic technology for entrepreneurship (see Sect. 4), should be mentioned. In particular, in textile processing bleaching can be performed with low bleach concentration and using H_2_O_2_ produced through water sonolysis [62]. Antibacterial nanoparticles (ZnO and CuO NPs), also generated by cavitation, are then embedded into textiles yielding a new class of antibacterial clothing suitable for clinical use against opportunistic pathogens. Likewise, a Japanese company (Cosmo Engineering Co. Ltd) was perhaps the first in investing in a pilot plant for the production of biodiesel (5,000 tons/year), based on palm oil transesterification, using two ultrasonic reactors with different volumes, each designed with a series of push–pull transducers inside [63].

## 3 Pedagogical approaches

Neither sonochemistry as a whole discipline, nor cavitation and its consequences are usually covered in a broad range of curricula in physics, chemistry, chemical engineering, or life sciences. Practical seminars, taking no more than 3-h sessions could augment the pedagogical aspects in both preliminary theory and experimental skills, thus enabling a deeper understanding of essential concepts. Despite the availability of low-cost instruments and numerous educational papers illustrating the wide applicability of sound waves, the adoption of ultrasound in lab courses is far from standard practice.

For undergraduate students, a solid background in physico-chemical principles underpinning their discipline is essential. Equally important, a good understanding of statistics analysis and error propagation is key to collect and interpret data. A range of active teaching methodologies involving practical and project/problem-based learning would benefit the application of practice-based sonochemistry teaching [64]. A constructionist approach, where knowledge and learning proceeds through experimental skills and activities, provides a suitable framework for beginners [65]. Advanced students can benefit from more heuristic approaches by applying previous knowledge to problem solving and decision making. A midway pedagogical approach is probably process-oriented guided inquiry learning, allowing students to construct knowledge through an iterative route of model exploration, concept invention (i.e. constructing an idea), and application (or extrapolation) of the idea to a new context [66]. Thus, the ultimate goal is to promote critical thinking with minimal instructor guidance [66]. Inquiry-based learning is facilitated by computer-aided approaches as shown in process intensification [67], which can include both conventional numerical simulations and emerging machine learning or artificial intelligence tools [68], [69], [70], [71]. Sonication, conceptualized as a kind of turbulent regime in which oftentimes acoustic cavitation occurs, cannot be fully understood without the aid of CFD. To these latter, considered as the mere fluid dynamics of flow patterns, shear rates, and turbulence in the liquid, should be coupled to other types of models considering several phenomena associated with ultrasound propagating in a liquid. These include eave propagation models, cavitation modeling (acoustic cavitation often attenuates shear rates), thermoacoustic modeling, particle dynamics, and chemical reaction kinetics. Multiphysics software, such as COMSOL, offer integrated modules to perform a compelling simulation of the systems to study.

The importance of demos and laboratory experiments in teaching sonochemistry and its applications has been repeatedly addressed [3], [5], [6], [72], [73], [74], [75]. Similarly, research-guided education has also been employed by numerous groups to boost sonochemistry itself as noted in a list of courses [27], [28]. Pedagogical goals imply a reference to interesting and often unusual effects of sonication, but also to expand the curricular coverage of textbooks in high schools and universities, where thermal, photochemical, or electrochemical applications are chiefly mentioned. The benefits are manifold in terms of interest and motivation, cognitive skills, and inter/multi-disciplinary learning. Moreover, regular symposia and summer schools represent driving forces attracting graduate and predoctoral students, besides workshops leading to white papers [27], [28].

### 3.1 Chemical dosimeters: first encounter with measurements

Box 1 collects a series of well-established experiments, suitable for students majoring in chemistry and chemical engineering, and/or related interface domains, environmental science in particular. Most, if not all, can be successfully conducted with “simple” ultrasonic baths (see Video1, Video 2). It is noteworthy that students do not need to be exposed to cavitation as primary concept, thus emphasizing the constructionist approach. Eye-catching experiments evidence the physical role of ultrasound waves, as portrayed by hole creation after dipping a kitchen aluminum foil into the bath; rapid dispersion of solids in liquids; emulsification of immiscible liquids; accelerated crystallization, as well as liquid degassing or ink removal, all echoing the science in everyday life [3], [6], [72], [76]. A spectacular foaming fountain can be generated by the sudden degassing of an uncapped carbonated beverage exposed to ultrasonic energy [77].

Box 1. Selected educational examples of lab experiments aided by sonication.

**Table:** 

*Lab demos/experiments*	*Concepts / skills*
Erosion/dispersion (Al foil; chalk in water) [3], [6], [74]	Mechanochemistry, thermodynamics, kinetics
Sonocrystallization (CuSO_4_·5H_2_O) [72]	Nucleation, kinetics, solid-state chemistry
Liquid degassing (soda fountain) [74], [77]	Henry’s law, solubility
Emulsification (water-methyl cyclohexane) [74]	Mechanical effects, interfacial phenomena
Lithium and Grignard reagents [4], [78], [79]	Metal-carbon bonding, cleaning effect, reaction mechanism
Organic synthesis (Diels-Alder) [76]	Reaction mechanism, home-made/modified sonoreactors
Degradation of chlorinated hydrocarbons [80]	Reaction mechanism, environmental remediation
Biodiesel synthesis [81]	Green chemistry, reaction mechanism
MOF synthesis [82]	Green chemistry, multicomponent synthesis
Luminol chemiluminescence [83], [84]	Kinetics, thermodynamics, reaction mechanism
Cleaning of delicate objects,e.g. 3D parts [85] 2D material exfoliation [35]	Mechanical effects, interfacial phenomena
Pd/Al_2_O_3_ catalyst in Cavitation intensifying bags (CIB) [86]	Advanced oxidation
Organic and hydroxy radical [48], [50], [87] dosimetry	Analytical chemistry, kinetics

However, training in ultrasonic power measurement is mandatory in introductions to the use of power ultrasound, a key point already mentioned (Sect. 2.2). This enables a quantitative approach to sonochemistry that can be employed from freshmen courses on. Along with calorimetry, ideal for baths, to measure the power entering a reaction, chemical dosimeters monitoring the sonochemical formation of a chemical species, can be applied to any insonated system. The most conventional dosimeter to characterize the production of free radicals is the liberation of triiodide ions from KI by sonochemically generated hydrogen peroxide. This venerable *Weissler reaction* is considered to be a relative measure of cavitation performance [88]. Transient cavitation leads to the decomposition of water vapor into HO and H radicals, equation [4]. Iodide ions are then oxidized by hydroxyl radicals in liquid phase, equation (5). In the absence of a radical scavenger, OH radicals form H_2_O_2_ by recombination in either gas phase or at the liquid–gas interface, equation (6).(4)
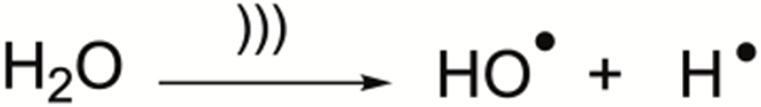
(5)
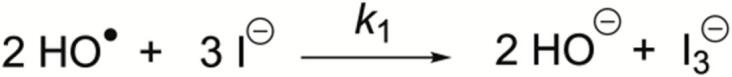
(6)
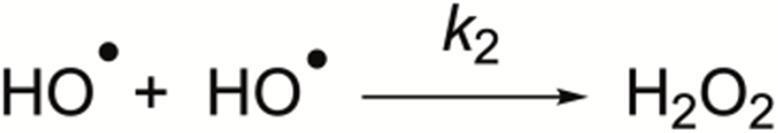


The rate constants of reactions [5] and [6] are similar and high, *k*_1_ = 1.1 x 10^10^ L/mol·s and *k*_2_ = 6.2 x 10^9^ L/mol·s. The concentration of triiodide ions (I_3_^−^) can easily be determined by UV–Vis spectrophotometry at λ = 355 nm. Aided by a Beer-Lambert plot, it is possible to calculate the concentration of H_2_O_2_ and to deduct the concentration of HO radicals [89]. When a probe is employed, the Weissler dosimetry is usually carried out from a 0.1 *M* KI solution. A 10 mL volume irradiated at 20 kHz for different reaction times at 20 °C using a cooling system, and triplicated under identical conditions, gives rise to a good straight line when absorbances (and hence, I_3_^−^ concentration) are plotted against time. To ensure reproducibility, ultrapure water is required and all of the glassware used should be scrupulously clean. Sonochemical efficiencies are also dependent on frequency and reactor configuration [90].

The so-called Fricke dosimetry represents another popular dosimeter, in which Fe(II) ions are oxidized to Fe(III), which are determined by photometry at λ = 304 nm [91]. A typical procedure involves the use of (NH_2_)_2_Fe(SO_4_)_2_ and NaCl dissolved in diluted H_2_SO_4_ solution and subjected to sonication. The number of HO radicals generated is approximately equal to one-quarter the amount of produced Fe(III) ions. Radical species can also be monitored by the therephthalate dosimetry [92], [93] [X5, X6], where hydroxyl radicals react with terephthalic acid in alkaline solution, buffered with phosphate (pH ∼ 7.4), yielding highly fluorescent 2-hydroxyterephthalate, equation [7]. Less usual dosimetries involve generation of nitrite and nitrate anions, which are determined by ion chromatography. Such species arise from radical oxidation of aqueous solutions containing dissolved N_2_ and O_2_ under sonication, with concomitant pH evolution evidencing the formation of nitrous and nitric acids [94]. Such research studies are interesting in terms of comparing the efficiency of chemical dosimeters for hydroxyl radical production at different frequencies, usually higher than those operating in standard baths and probes. Students and newcomers can ignore such specialized considerations, while capturing the essential point, i.e. how ultrasound induces chemical phenomena that can be quantified(7)
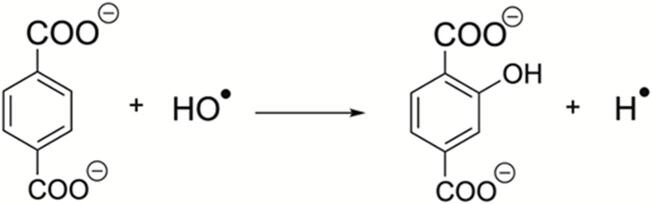


### 3.2 Sonochemical demonstrations at educational level

Dosimetry and other visual demos noted above inform students and novices about an intense form of energy when power ultrasound propagates through the liquid. Understanding chemical activation does actually require a few ideas on bubble collapse, because acoustic energy *per se* cannot break chemical bonds in the way high-frequency light does. Thus, initiation of otherwise reluctant metalation reactions constitutes a hallmark of modern sonochemistry, as formation of lithium or Grignard organometallics is accelerated under sonication without requiring dry solvents or inert atmosphere [4], [78], [79]. Interestingly, this sort of experiment can safely be carried out in a cleaning bath. Students can also be invited to determine before the acoustic efficiency of the ultrasonic device by comparing two different dosimeters, such as calorimetry and the Weissler reaction.

Another distinctive and easily demonstrative example is the “cleaning effect” of ultrasound on surfaces, thereby removing impurities from metal or 3D printed surfaces [85]. Such effect can easily be understood, and these experiments also facilitate the investigation of reaction mechanisms, as the rapid formation of metal–carbon bonds could occur either by a conventional polar mechanism or electron transfer after bubble collapse near the surface. A similar reasoning can be invoked for homogeneous reactions involving labile bonds, such as the enhanced degradation of halogenated hydrocarbons in aqueous solutions by sonication [80]. This type of experiments is highly instructive as low molecular weight chlorinated compounds can be degraded upon irradiation in aqueous solutions and releasing HCl; the lowering of pH can be visually detected by means of acid-base indicators present in solution. We checked the reported case with minimal modification (it works better with more concentrated solutions) and found it to be reproducible. The lab experiment was performed by first- and second-year undergraduate students, who had not received previous training in cavitation (baths were not calibrated either). However, the interaction with sonic energy for the first time was extremely positive and reinforced other concepts in bonding and thermodynamics. A deeper insight into the same experiment was gained by master students after receiving a 3 h-module in sonochemistry.

Consistent with current green chemistry goals, lab experiments involving the acceleration of biodiesel production (i.e. ultrasonic-aided transesterification) [81] or ultrasonic-assisted synthesis of metal–organic frameworks (MOFs) [82] are likewise pedagogical in context. Here, the mechanical activation (enhanced mass transfer) caused by ultrasonic agitation in heterogeneous processes constitutes a salient feature.

**Fig. 9:**
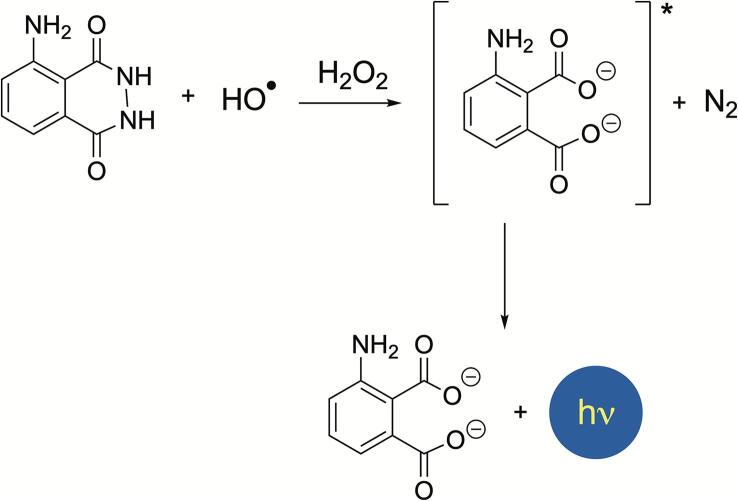
Luminol is oxidized in basic aqueous solution in the presence of a strong oxidant such as hydrogen peroxide. The latter, sonochemically generated by combination of HO radicals, leads to enhanced chemiluminescence emitting visible blue light flashes. (For interpretation of the references to colour in this figure legend, the reader is referred to the web version of this article.)

Advanced students will benefit from the synergy between sound activation and light emission, as exemplified by the enhanced chemiluminescence of luminol in alkaline conditions. This appealing *sonoluminescence* experiment reveals the extreme conditions created by bubble collapse accompanied by emission of photons. As shown in Fig. 9, Fig. 3-aminophthalhydrazide (luminol) oxidizes in the presence of HO radicals into 3-aminophthalate anion in an excited state, which deenergizes through visible blue light emission at λ = 430 nm. This method cannot be regarded as dosimeter, as it is not quantitatve. It allows however a precise mapping of acoustically active zones [83], [84]. The effect is enhanced further by addition of halomethanes, which illustrate well the role of water sonolysis and sonofragmentation of carbon-halogen bonds. Students can use their own mobile phone camera as visual detector of this phenomenon.

## 4 Education in entrepreneurship for sonochemists

A typical Science Technology Engineering Mathematic (STEM) student in the classroom right now is learning about technologies that might not be sufficient to tackle the ever fast-changing challenges of our time, let alone facing the hardships of persuading, or even understanding the needs of society. Simply put, it is too much to learn in a four-year BSc/BEng, and it only incrementally improves with extra degrees, e.g., MSc/MEng and PhD. We know that the Ultrasonics Sonochemistry community is diverse, and there is untapped potential to increase its impact for problems that are societally relevant. We consider that there is potential for sonochemists to contribute to impactful solutions in both academia and companies.

There is, providentially, a quality or ‘ingredient’ that some people possess through personality or through upbringing and is also taught in a handful of STEM study programs: Empathy, i.e. “the ability to understand or predict the perspectives of others, and from that understanding, accurately identify what the needs and desires of that person or group are” [95]. We are proponents of a simplified conceptual framework for the training of engineers to be more innovative and entrepreneurial. It is based on three core components or ingredients: *knowledge, persuasiveness, and empathy*. We believe that these ingredients can be used to initiate a necessary shift in how students are educated in fields of science, technology, engineering, and mathematics, to which Ultrasonics Sonochemistry belongs. Developing persuasiveness and empathy as durable skills can be framed in the universally used Challenge Based Learning context and a six-step procedure has been proposed as a guideline to turn the knowledge, persuasiveness, and empathy framework into actionable items. We join the efforts to explicitly teach the importance of durable people-oriented skills in combination with technical courses, ideally spreading the focus over the whole curricula.

Considered by some as ‘soft’, we should include Empathy and Persuasiveness in the ‘durable’ skills toolbox, because what we learn as social skills outlive any technology development [2]. But how can we teach it? And once we find how to teach it, can we also do it outside the classical educational settings? Can it be done via online tools during the next pandemic-induced restrictions? As educators, we must define how to provide students with knowledge – or the chemistry fundamentals – but also much needed interactions with other disciplines to help them gain durable skills, crucial to becoming successful scientists or entrepreneurs of the future [18].

Sonochemistry lends itself well to fulfilling this link between entrepreneurship and education. One way could be assigning projects that require students to identify real-world challenges that can be addressed using sonochemistry. The fields to be tackled include materials’ synthesis, metal processing, soil and water remediation, and materials engineering. Students are prompted to test not only their technical knowledge but also their persuasiveness in proposing an ultrasound-based solution to solve a challenge in these very diverse fields. The case-studies could be proposed to successful entrepreneurs who have utilized sonochemistry for innovative solutions.

Luckily, there are several cases of entrepreneurial approaches using sonochemistry, from industry and academic founders. In our courses and lectures, we argue that there are other motivations besides increased income when starting an entrepreneurship path [18]. Moreover, there is a positive shift in recognizing that academicians can be entrepreneurs without abandoning academia. In the educational context, similar to commercialization and entrepreneurial activities, there is an equivalent approach of making demonstrations of a given product with ultrasonic equipment which is relatively easy. If we consider portable ultrasonic baths or horns, they can be used to show in real time a given process (e.g., Video 3 and Videos 1 and 2, Section 3.1) A non-exhaustive list of companies that have made innovations and taken risks to go beyond the mere curiosity-driven academic or scientific explorations is also given in Section 2.4.

## 5 Conclusions and outlook

The adoption of sonochemistry as an educational tool in chemistry, chemical engineering and other disciplines easily conveys the advantages of a widely available method that can “activate” several processes. These processes include chemical reactions, physico-chemical processes (such as crystallization), or extractions. The fields of applications range from the production of commodity reagents and materials, reduction of waste, mitigation of environmental impact, or real-time monitoring in medicine, construction, and quality control.

The drive to develop better models and tools to incorporate sonochemistry into education will also require an assessment of a series of learning points: (1) acquisition of knowledge of macroscopic parameters that can be accurately measured, as well as microscopic consequences of cavitation at molecular level in a given experiment; (2) choice of appropriate ultrasound devices and experimental scales tailored for specific purposes; (3) consideration of rigorous standardized procedures to determine the influence and reproducibility of the ultrasonic activity; (4) assessment of concerns related to human safety and environmental fate of ultrasound-based protocols.

The integration of sonochemistry in undergraduate and postgraduate curricula can easily be accomplished through transferring basic knowledge and training with the methods described in this manuscript. However, continuous improvement is required on both pedagogy and experimental-wise to reach standardized methods that allow for a comparison among studies, and for stimulating feedback in the classroom and laboratory.

Lastly, we have provided a link between academia, industry, and society, which we encourage the educators to explore with their students. In particular, we see an urgency in highlighting the value of entrepreneurial activities, based on nurturing a balance between knowledge, persuasiveness, and empathy.

## CRediT authorship contribution statement

**David Fernandez Rivas:** Writing – review & editing, Writing – original draft, Supervision, Investigation, Formal analysis, Conceptualization. **Pedro Cintas:** Writing – review & editing, Writing – original draft, Conceptualization. **Jarka Glassey:** Writing – review & editing, Writing – original draft, Conceptualization. **Daria C. Boffito:** Writing – review & editing, Writing – original draft, Conceptualization.

## Declaration of competing interest

The authors declare the following financial interests/personal relationships which may be considered as potential competing interests: David Fernandez Rivas reports a relationship with BuBclean that includes: board membership and equity or stocks. D.F.R. is a founder and a scientific advisor of BuBclean, a Dutch start-up company that commercializes the BuBble Bags (Cavitation Intensifying Bags - CIB) and operates in the ultrasonic cleaning solutions market. If there are other authors, they declare that they have no known competing financial interests or personal relationships that could have appeared to influence the work reported in this paper.
